# Psychological Effects of False-Positive Results in Expanded Newborn Screening in China

**DOI:** 10.1371/journal.pone.0036235

**Published:** 2012-04-27

**Authors:** Wen-Jun Tu, Jian He, Hui Chen, Xiao-Dong Shi, Ying Li

**Affiliations:** 1 Center for Clinical Laboratory Development, Chinese Academy of Medical Science and Peking Union Medical College, Beijing, People's Republic of China; 2 China Rehabilitation Research Center, Beijing, People's Republic of China; 3 Department of Pediatrics, Beijing Haidian Maternal and Child Health Hospital, Beijing, People's Republic of China; Indiana University, United States of America

## Abstract

**Objectives:**

As more families participate expanded newborn screening for metabolic disorders in China, the overall number of false positives increases. Our goal was to assess the potential impact on parental stress, perceptions of the child's health, and family relationships.

**Methods:**

Parents of 49 infants with false-positive screening results for metabolic disorders in the expanded newborn screening panel were compared with parents of 42 children with normal screening results. Parents first completed structured interview using likert scales, closed and open questions. Parents also completed the parenting stress index.

**Results:**

A total of 88 mothers and 41 fathers were interviewed. More mothers in the false-positive group reported that their children required extra parental care (21%), compared with 5% of mothers in the normal-screened group (P<0.001). 39% of mothers in the false-positive group reported that they worry about their child's future development, compared with 10% of mothers in the normal-screened group (P<0.001). Fathers in the false-positive group did not differ from fathers in the normal-screened group in reporting worry about their child's extra care requirements, and their child's future development. Children with false-positive results compared with children with normal results were triple as likely to experience hospitalization (27%vs 9%, respectively; P<0.001).

**Conclusions:**

The results showing false-positive screening results may affect parental stress and the parent-child relationship. Parental stress and anxiety can be reduced with improved education and communication to parents about false-positive results.

## Introduction

Expanded newborn screening (NBS) using tandem mass spectrometry (MS/MS) to identify more than 30 biochemical genetic disorders is an important advance in early disease detection. It has greater sensitivity than past screening methods and allows for presymptomatic detection and identification of metabolic disorders [1]. However, expanded newborn screening has led not only to an increase in positive identifications but also to a dramatic increase in the overall number of out-of-range results, of which the majority are confirmed to be false positives after further testing [2]. Generally these results are not laboratory mistakes but rather are transient findings or indications of variant or carrier status. The current overall risk of a false positive result for expanded NBS in the United States is estimated to range between 1/1,500 to 1/3,600 [3]–[4]. False-positive screening results have been associated with increased anxiety and stress in parents of infants who require follow-up testing, even after the infant's good health is confirmed [5]. Studies also reported long-term negative effects including alterations in perceptions of their infant's health, an increase in the number of emergency room visits, and hospitalizations for the infant [5]–[8].

This report firstly describes china parents' responses to false-positive newborn screening results among a cohort of children born after January 1, 2008, when voluntary expanded newborn screening began in Beijing. The psychological effects of false-positive have not been studied in Chinese population before.

## Methods

### Enrollment and Study Procedures

Mothers and fathers of children with false-positive newborn screen results, defined as the initial result being abnormal or inconclusive for any of 35 biochemical disorders, were invited to participate after a referral was made for additional confirmatory testing. The authors contracted with a screening center, Center for Clinical Laboratory Development, Chinese Academy of Medical Science, which conducts newborn screening for more than 80% of birthing hospitals in Beijing, to recruit and interview parents of infants with false-positive newborn screen results. This was a comparative cross sectional study. Participants were enrolled by this laboratory between 2008 and 2009. Parents of children with false-positive results were sent a recruitment letter with a reply paid envelope more than 6 months after the diagnosis of a metabolic disorder had been ruled out. This inviting letter included, a short questionnaire (2 copies), two written informed consent (study purpose, methodology of the protocol, risks, direct and indirect potential benefits, the right to withdraw, duration of participation, possibility of alternative treatments, voluntariness) [9], and a letter of explanation the study.

Parents of twins and triplets were sent a single letter. Parents who did not “opt out” by returning a response card indicating a preference not to be contacted were called to participate in a telephone interview [8], [10]. Although both parents were invited, it was acceptable if only 1 parent participated.

The comparison group for the false-positive cohort consisted of parents of 6- to 12-month-old children with normal screening results, selected randomly from the screening center database.The storage NBS card contain the date of birth, birth weight, parents' names, birth hospital, and address. All recruitment occurred between January 1, 2008, and December 31, 2009. All participants completed the study questionnaire once. Additional follow-up interviews were not included in the study design.

Exclusions included parents of children who had died and parents of newborns whose birth weight was 2500 g. or gestation less than 32 weeks [10]–[11]. The latter exclusion avoided recruitment of parents of premature newborns, who frequently experience transient initial newborn screening abnormalities. Approval for this study was obtained annually from the institutional review board of Center for Clinical Laboratory Development, Chinese Academy of Medical Science and Peking Union Medical College. Written informed consent was obtained from all participants involved in our study. By design, the number of false-positive participants exceeded the number of participants in the normal group. Statistical methods that did not require balanced sample sizes were selected.

### Data Collection Instrument

Complete details of data collection have been reported previously [10]–[13]. Parents responded to a structured questionnaire (study instrument) using likert scales and closed and open questions. This sought to determine from the parents (a) whether they would have the test performed again for another child, (b) in the first 6 months of life, whether [they] had ever taken [their] child to inpatient, and the dates of such visits; how many times did your babies visit a primary care physician, and the dates of such visits, (c) whether they knew the screening could lead to false-positive result when they took part in expanded newborn screening, (d) whether their child required extra parental care, (e) whether they fear that their child might be developmentally delayed, or experience a false-positive screening result as a significant threat to the child's well-being, (f) reasons for repeat screen. Parents provided short answers or ratings on a 5-point Likert scale. The questionnaire also assessed sociodemographic factors (age and parity of the mother, level of education, income, and their site of residence) and one open ended question, “what change can be made in the expanded screening process”. The same interview was given to both groups of parents, but only parents in the false-positive group were asked questions about (f).

Parents next completed the parenting stress index (PSI), short form [8], [14]. This is a 36-item questionnaire that provides a total stress score and 3 subscale scores, namely, parental distress, parent-child dysfunctional interaction, and difficult child. The normal range for total stress scores is 55 to 85, with scores of 85 considered to be in the clinical range in which treatment may be necessary. The PSI also provides a defensive responding index, which is an internal index of validity based on the parent's responses. Scores of 10 for this index indicate that the validity of the total stress and subscale scores is questionable [8], [14]. All of the items in the PSI used in our sample were translated into Chinese by the first author and back translated into English by a professional translator. The first author followed the strictest translation procedure: back translation, informal interviews, pre-test, and item analysis, to ensure cultural equivalence. The Chinese version of the PSI had high reliability and predictive validity. The cronbach's *a* coefficient in this study was 0.92.

### Data Analyses

Statistical Package for Social Science, SPSS version 15.0 (SPSS Inc., Chicago, IL, USA) was used for data entry and analysis. Descriptive statistics such as means and standard deviation (SD) for age of mothers and frequency and percentages for categorical variables (such as race, gender of neonate, number of child, family income) were determined. The characteristics of children, parents, and families in the false-positive group were compared with those in the normal-screened group by using the student's unpaired t-test for continuous and scale variables and fisher' exact test for dichotomous variables. Student's unpaired t-test was also used to compare the PSI scores between different groups. The number of hospitalizations occurring before 6 months of age was compared for the 2 groups by using Poisson regression. For the PSI, subjects who failed the defensive responding index (scores of 10) were dropped from the analyses. The result of open ended questionnaire was analysis by frequency and percentages. All P values were2-sided, and values of <0.05 were considered significant [8].

## Results

### Sample

The sample included parents of 49 children with false-positive newborn screen and 42 children with normal newborn screen results. A total of 88 mothers (47 false-positive and 41 normal-screened) and 41 fathers (23 false-positive and 18 normal-screened) were interviewed. For 38 infants (21 false-positive and 17 normal-screened), both parents responded. The number of enrolled families divided by the number of families contacted determined the participation rates, which were 48% for the false-positive group and 42% for the normal-screened group.

As noted in Table 1, the false-positive group was similar to the comparison group in terms of parent age, gender, birth order, ethnicity, marry. In the false-positive group, children were older at the time of evaluation (mean: 12.4 months, SD: 3.2 months) compared with the normal screened group(mean: 6.7 months; SD: 1.2 months; P<0.001). The false-positive group was of lower economic or education status, compared with the normal-screened group (P<0.001). In additional, according to parental report, the median age of the infant's diagnosis was confirmed was 21 days (range: 7–94 days).

**Table 1:** Comparison of demographic profiles of the respondents.

variable	False-Positive (N = 49)	Normal-screened (N = 42)	p^a^
Parents' age, mean (SD)^b^, mo	29.7 (6.32)	28.9 (6.15)	0.75
Child male, n (%)	26 (53)	22 (52)	0.80
Child first-born, n (%)	35 (71)	33 (79)	0.22
Chinese race, n (%)	47 (96)	40 (95)	0.83
Married families, n (%)	46 (94)	41 (98)	0.68
Child age at evaluation, mean (SD), mo	12.4 (3.2)	6.7 (1.2)	<0.001
Family income (RMB/Year)^c^, n (%)			
28,000 or less	19 (40)	12 (29)	<0.001
28,000–88,000	17 (36)	15 (37)	0.76
88,000 or more	11 (24)	14 (34)	<0.001
Education background^d^, n (%)			
High school or less	46 (68)	30 (53)	<0.001
College or more	22 (32)	27 (47)	<0.001

a Fisher's exact test for dichotomous variables and Student's unpaired t-test for continuous variables.

b N = 70 in the False-Positive group, 59 in the Normal-screened group.

c N = 47 in the False-Positive group, 41 in the Normal-screened group; 1 U.S. dollar = 6.311 RMB.

d N = 68 in the False-Positive group, 57 in the Normal-screened group.

### Parental Stress and Parent-Child Relationship

Although parents in the 2 groups reported both worry about their child's health, more mothers in the false-positive group reported that their children required extra parental care (21%), compared with 5% of mothers in the normal-screened group (P<0.001). Thirty-nine percent of mothers in the false-positive group reported that they worry about their child's future development, compared with 10% of mothers in the normal-screened group (P<0.001). Fathers in the false-positive group did not differ from fathers in the normal-screened group in reporting worry about their child's extra care requirements, and their child's future development. In additional, thirty-seven percent of parents in the false-positive group reported that they child have visited a primary care physician in the first 6 months of life, while 15% parents in the normal-screened group (P<0.001). The child hospitalizations during the first 6 months of life among false-positive group was 0.27, compared with 0.09 in the normal- screened group (P<0.001).

As shown in Table 2, mothers in the false-positive group reported higher overall stress on the PSI than did mothers in the normal-screened group. 17% of mothers in the false-positive group (n = 8) but no mothers in the normal-screened group scored in the clinical range. The differences between groups were more pronounced on the total score, parent-child dysfunctional interaction subscales, and difficult child subscales than on the parental distress subscale. Fathers in the false-positive group also registered higher overall stress on the PSI than did fathers in the normal screened group, especially on the total score (P = 0.01), and difficult child subscales (P<0.001).

**Table 2:** Impact on the family: PSI scores for False-Positive and Normal-screened group.^a^

variable	PSI score, mean ± SD		P value^b^
	False-Positive (44 mothers, 22 fathers)	Normal-screened (40 mothers, 17 fathers)	
Total score			
Mothers	75.5±13.2	60.7±10.1	<0.001
Fathers	72.7±14.6	66.1±11.9	0.01
Parental distress subscale			
Mothers	29.6±5.2	26.6±6.2	0.04
Fathers	28.1±5.7	27.1±6.6	0.82
Difficult child subscale			
Mothers	25.7±5.6	18.5±4.9	<0.001
Fathers	25.1±6.2	21.2±5.5	<0.001
Parent-child dysfunction interaction subscale			
Mothers	19.9±5.5	15.6±3.6	<0.001
Fathers	19.5±6.9	17.8±4.2	0.62

a Higher scores indicate higher stress; only PSI scores for subjects whose defensive responding index was >10 were included in the analysis [16];excluded were 4 mothers (3 in the false-positive group) and 2 fathers(1 in the false-positive group).

b Student's unpaired t-test.

### Parental Knowledge to the Screening Process

As shown in Table 3, 55% (n = 26) of mothers and 50% (n = 11) of fathers knew the correct reason for their child needing a repeat screen. Mothers in the false-positive group who knew the correct reason for the repeat screen reported lower stress levels on the PSI, including the total score, difficult child subscales, and parent-child dysfunctional interaction subscales (Table 4). Fathers who knew a correct reason for the repeat screen did not exhibit lower stress levels on the PSI (P = 0.09).

**Table 3:** Parents response to reason for repeat screen in false-positive group.

Parent report of reasons	Response, %	
	Mothers (N = 47)	Fathers (N = 22)
Correct responses	55	50
Test indicated metabolic disorder	23	23
Initial test result was abnormal	17	18
Test inconclusive	15	9
Inaccurate responses	33	23
Not enough blood collected	15	14
First test had a mistake or was lost	12	9
Repeat screen is routine	6	0
Other	12	27
Cannot remember	8	18
Nothing specific	4	9

**Table 4:** PSI scores for parents in false-positive group according to self-reported reason for repeat screen.^a^

variable	PSI score, mean ± SD		P value^b^
	Correct reason given (26 mothers, 11 fathers)	Correct reason not given (21mothers, 11fathers)	
Total score			
Mothers	68.3±11.4	77.2±13.1	<0.001
Fathers	72.3±12.5	73.2±12.9	0.11
Parental distress subscale			
Mothers	26.2±5.1	27.6±6.5	0.32
Fathers	27.3±5.2	25.9±4.9	0.76
Difficult child subscale			
Mothers	23.3±6.1	26.5±5.4	0.02
Fathers	25.8±7.2	24.6±5.5	0.05
Parent-child dysfunction interaction subscale			
Mothers	18.8±5.6	23.1±5.6	<0.001
Fathers	19.2±6.4	22.7±5.8	0.01

a Higher scores indicate higher stress; only PSI scores for subjects whose defensive responding index was >10 were included in the analysis [16].

b Student's unpaired t-test.

Parents in the false-positive group reported a lower tolerance of false-positive results. Ten (14%) parents in the false-positive group reported that they would not have the test performed again for another child, while no parents in the normal-screened group. 28% parents (19 in false-positive group, 17 in normal-screened group) reported that they didn't know false-positive when they began participate in screening. In the last open-ended question: “what change can be made in the expanded screening process”, fifty-six percent (n = 72) of parents expressed a need for more information about newborn screening and false-positive results, 16% (n = 21) of parents voiced that providers should provide clearer explanations of the reasons of repeat screening.

## Discussion

Expanded newborn screening programs have expanded dramatically in the past decade. The main risks are related to false positive results and results with ambiguous implications for treatment-risks. Our results indicate that a false-positive result from an expanded newborn screening test can induce some parents to experience stress and affect parents' perceptions of their child's health, and the parent-child relationship. This finding is expressed by parents' higher overall stress on the PSI, more than four times required extra parental care, 3 times the number of children hospitalized, longer hospital stays in the false-positive group compared with the normal screen group.

False-positive screening results have been associated with increased anxiety and stress in parents of infants who require follow-up testing, even after the infant's good health is confirmed. The true impact of false-positive newborn screening tests is just beginning to be well described. Early screening programs for phenylketonuria (PKU) showed poor parental understanding of false-positive results and a tendency for parents of such children to perceive their children as medically vulnerable [15]. Studies suggest that some parents of these infants remain anxious about their child's health, perceive the child as unhealthy, and, as a consequence, treat the child differently even after a result is deemed a false-positive finding [4], [16]–[17]. More than one third parents still have concerns about the health of their infant, on average, mothers report more stress [4]. These findings are consistent with our study that has shown 39% mothers experience a false-positive screening result as a significant threat to the child's well-being. Other studies on the impact of acute illnesses among children identified the “vulnerable child syndrome” [18]–[19].Those studies applied the vulnerable child syndrome to include (1) a condition or even a “non-disease” (eg, false-positive result) in a child, (2) parents who misinterpret hat condition or its sequelae, and (3) parents who exhibit sustained unjustifiable anxiety about the child's vulnerability to future events [8].

More recent research has demonstrated associations between false positive results and mothers' perceptions that their children with false positive results require increased parental care, and a trend towards increased hospitalization [5].Studies have led to speculation that a false-positive result would be associated with increased outpatient and inpatient health care utilization in early childhood [20]–[23]. This might explain a trend toward an increase in infant hospitalizations during the first 6 months of life among false-positive children in our study (mean: 0.27 hospitalization VS 0.09 hospitalization; P<0.001). Earlier studies have found an association between false-positive newborn screen results and negative psychosocial effects [24]. This association was also documented in studies screening for hearing, cystic fibrosis, diabetes [25]–[28]. We hypothesized that such psychosocial effects could lead to parents perceiving children with false-positive test results as vulnerable which in turn may lead to increased health care utilization. “Our results are consistent with this hypothesis, despite other research reporting the contrary [12]–[13].

Studies have demonstrated that education of parents about false-positive results is lacking [29]. It is clear that even after routine NBS testing, a significant proportion of parents are confused about the meaning and reasons for repeat testing after an initial NBS test [8], [10]. Similarly, in our study, 48% parents of children with false-positive results did not know the correct reason for their child's follow-up testing. It may be that physicians do not communicate the false-positive result to families, all the knowledge about false positive come from parents themselves. This idea is supported by the fact that, as detailed elsewhere, in our study sample 28% parents reported that they didn't know false-positive when they began participate in screening. Researchers have consistently shown providers' ability to communicate about newborn screening is poor in both training and primary care settings [30]–[33]. Nonetheless, in a recent study of paediatricians in Massachusetts, 42% were less than comfortable talking about newborn screening test results with families [32].

The results of *Hewlett*'s review suggest that parental stress and anxiety can be reduced with improved education and communication to parents, specifically at the time of follow-up screening [5]. Physicians seem to be able to reduce parents' stress if they provide information about the process (as well as the false-positive results) of newborn screening, estimate the risk to the infant as low, or refer parents for additional information. Similarly, in our study, mothers who knew the correct reason for their child's repeat screening test experienced less total stress than did mothers who did not know. In additional, parents in our study suggested ways in which the process could be improved to reduce the influence of false-positive results, including provision of more information about newborn screening and false-positive results. They also suggested that providers should provide clearer explanations of the reasons of repeat screening.

This study has a number of limitations. The disparity in the children's ages between the false-positive and normal-screened groups could have biased results. However, the PSI used to measure parental stress, is considered age independent for small increments of age. It is possible that our study design, in which participants were interviewed 6 months after the resolution of the false-positive screening result, may not have fully captured the stress and anxiety experienced during the waiting period. In additional, sample sizes were small for both respondent groups. The samples were also geographically limited, potentially limiting the generalizability of our results. In the process of our study, the response rate was 45%, lower than other reported [10], [33]–[36]. This rate means that the finding should not be overinterpreted. These experiences relate largely to a single survey in one center. It cannot be assumed that they will apply to other center or population.

This study suggests that false-positive screening results may affect parental stress. This is especially true for parents who have not received adequate information about newborn screening. Therefore, Parental stress and anxiety can be reduced with improved education and communication to parents about false-positive results.
